# Supplementary material for: Thermodynamic Determinants in Antibody-Free Nucleic Acid Lateral Flow Assays (AF-NALFA): Lessons from Molecular Detection of Listeria monocytogenes, Mycobacterium leprae and Leishmania amazonensis
Source: Biomolecules. 2025 Oct 2;15(10):1404. doi: 10.3390/biom15101404 (PMC12562187; doi:10.3390/biom15101404)
Supplement: Supplementary file 1 [file biomolecules-15-01404-s001.zip › biomolecules-3891712-supplementary.pdf]

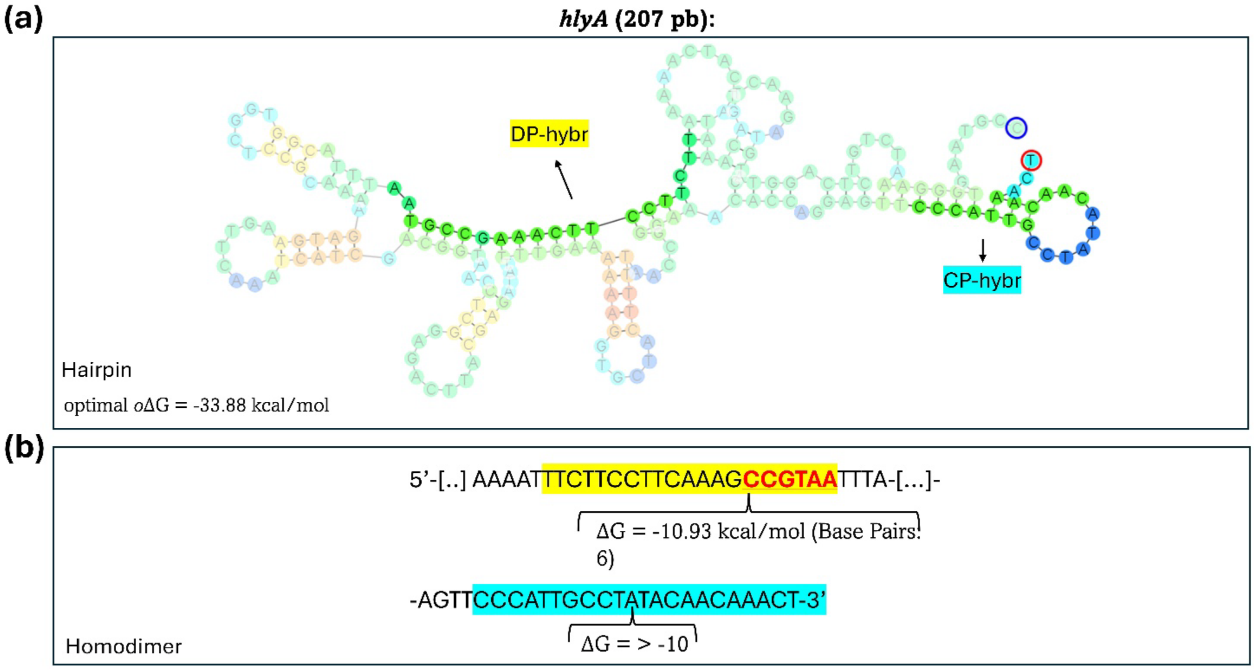

Supplementary Figure S1. In silico analysis of secondary structure and thermodynamic behavior of native *l. monocytogenes hlyA* ssDNA amplicons used in AF-NALFA. (a) Geneious® DNA Fold prediction of hairpin structures for *hlyA* (207 bp). Color gradients reflect thermodynamic stability (warmer colors = more stable base pairing). Only the regions corresponding to the hybridization sites of the detection probe (DP-hybr, yellow) and capture probe (CP-hybr, cyan) are shown clearly; the remaining sequence is blurred. (b) IDT OligoAnalyzer™ homodimer predictions. Yellow regions represent detection probe binding sites, and cyan regions indicate capture probe binding sites. Red-colored bases were used to compute site-specific Gibbs free energy (*site-specific*-ΔG, kcal/mol). Values below −10 kcal/mol suggest impaired accessibility. “[...]” denotes truncated sequence. Brackets indicate the number of paired bases.

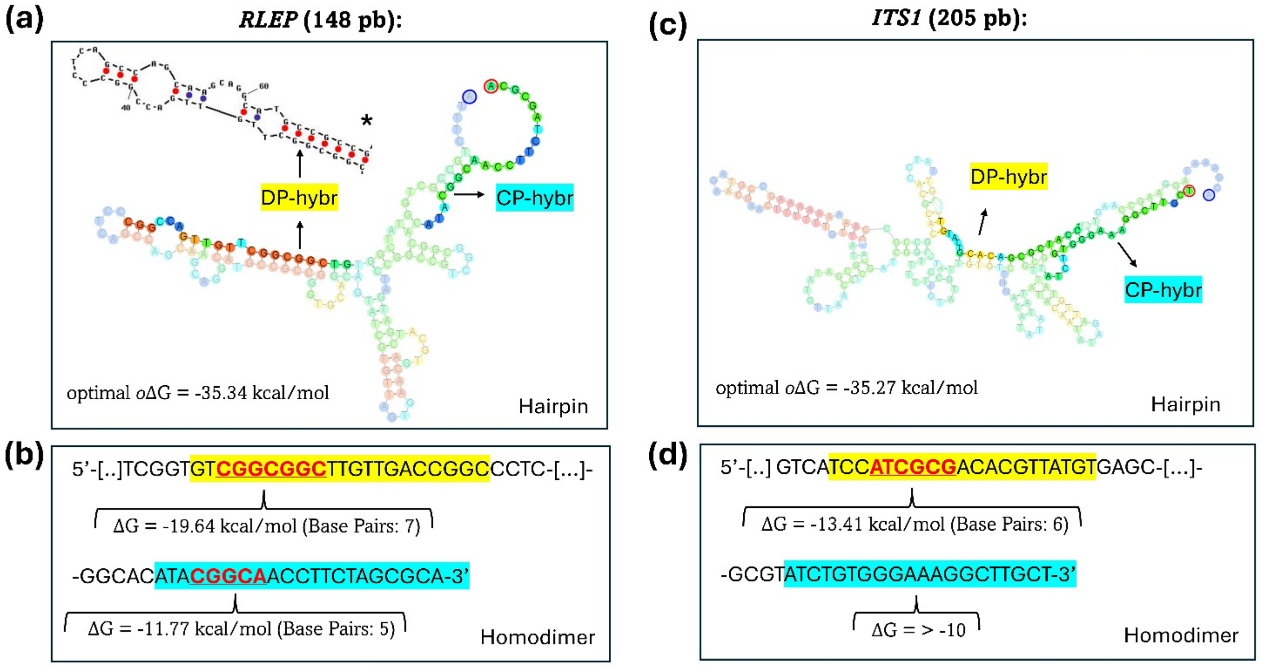

Supplementary Figure S2. Secondary structure and homodimer analysis of *M. leprae RLEP* and *L. amazonensis ITS1* native ssDNA amplicons. (a–c) Geneious® DNA Fold prediction of hairpin structures for *RLEP* (148 pb) and *ITS1* (222 bp). Color gradients indicate base-pairing stability, with warmer colors denoting greater structural constraint. Only the hybridization regions of the detection probe (DP-hybr, yellow) and capture probe (CP-hybr, cyan) are fully visible; other regions are blurred. In *RLEP*, an asterisk (*) marks a structural fragment from OligoAnalyzer™, showing nucleotides involved in secondary structures (red dots). (b–d) Homodimer formation predicted using IDT OligoAnalyzer™. Detection probe (yellow) and capture probe (cyan) binding regions are highlighted. Red-colored nucleotides represent the bases used to calculate *site-specific*-ΔG values. Sequences with ΔG higher than −10 kcal/mol were considered structurally accessible. "[...]" indicates partial sequence context.

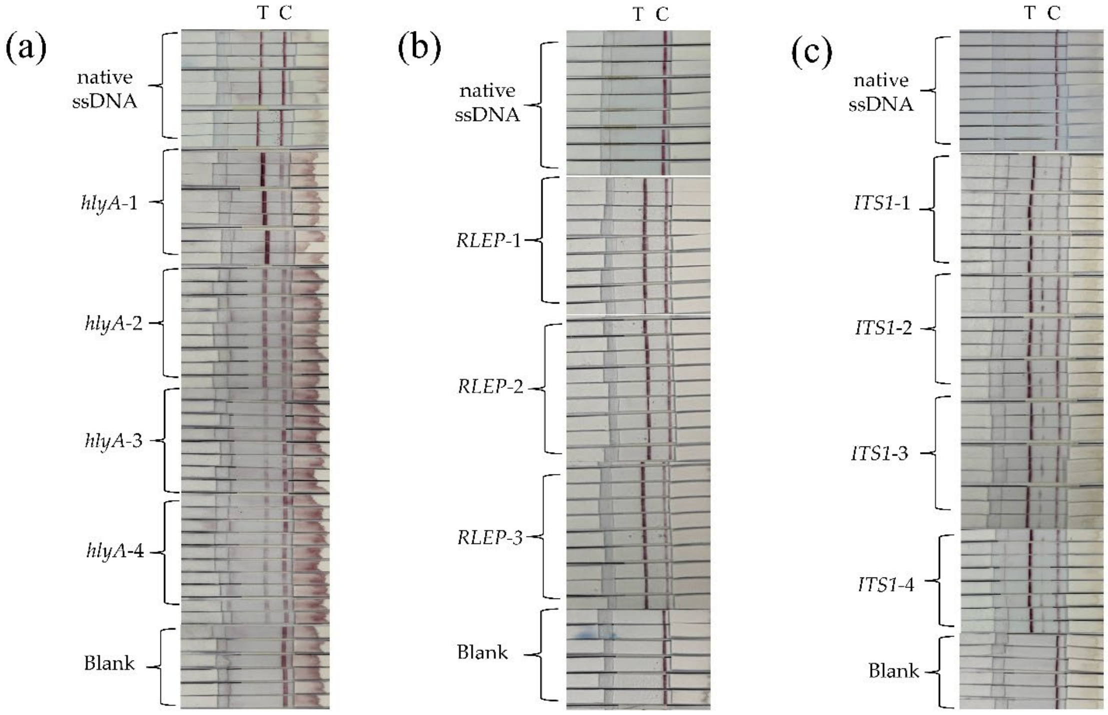

Supplementary Figure S3. AF-NALFA strips obtained with native ssDNA, synthetic truncated constructs and blank controls for (a) *Listeria monocytogenes hlyA*, (b) *Mycobacterium leprae RLEP*, and (c) *Leishmania amazonensis ITS1*. In all strips, the test line (T) is located on the left and the control line (C) on the right. In panel (c), some strips display three lines; in these cases, only the first line corresponds to the *ITS1* target and the third (rightmost) line is the control, while the middle line refers to another multiplex target and should be.

Supplementary Table S1. Sequences and thermodynamic parameters of native and synthetic truncated ssDNA amplicons used in AF-NALFA. Sequences correspond to native aPCR products and structurally modified oligonucleotides designed for *L. monocytogenes, M. leprae* and *L. amazonensis*. Hybridization regions for the detection probe (5' end) and capture probe (3' end) are underlined and separated by an asterisk (*). Italicized bases indicate the exact nucleotide sequence used for calculating site-specific Gibbs free energy (*site-specific*-ΔG) for homodimer prediction via IDT OligoAnalyzer™. Nucleotides shown in black, bold, enlarged font with orange-red glow represent bases predicted to remain structurally accessible (not involved in hairpin or homodimer formation) within the detection or capture probe regions based on *site-specific*-ΔG analysis. Nucleotides highlighted with green glow indicate hypothetical single nucleotide polymorphisms (SNPs), while bases highlighted with blue glow denote bases involved in homodimer formation with *site-specific*-ΔG values between –10 and –15 kcal/mol. Thermodynamic parameters were predicted using Geneious® and OligoAnalyzer™ tools, including free energy of optimal (F.E.O.) hairpin formation, homodimer stability, and *site-specific*-ΔG values (*s-s*ΔG). More negative ΔG values indicate increased structural stability and potential impairment of probe hybridization. All in silico analyses and structural predictions were performed at 25 °C.

Pathogen	Identification	Sequences (5'-3')	F.E.O.: hairpin Geneious®	F.E.O.: hairpin IDT™	F.E.O.: homodimer Geneious®	*s-s*ΔG – DP IDT™	*s-s*ΔG – CP IDT™
L. monocytogenes	native ssDNA (207 pb)	CCGTAAGTGGGAAATCTGTCTCAGGTGATGTAGAACTGACAAATATCATCAAAAATTCTTCCTTCAAAGCCGTAATT**TACGGTGG*CTCCG*CAAAAGAT**GAAGTTCAAATCATCGACGGTAACCTCGGAGACTTACGAGATATTTTGAAAAAAGGTGCTACTTTTAACCGGGAAACACCAGGAGTT**CCCA*TTG*CCTATACAACAAACT**	-33.88	-21.38	-74.09	-9.85	-3.90
	Truncated amplicon: *hlyA*-1	**TA**CGG**TGGCT**CC***GCAA*AAGAT*******C*CCATTGCCTATACAACAAACT**	-4.71	-3.69	-15.15	-7.04	-4.62
	Truncated amplicon: *hlyA*-2	**TA**CGG**TGGCT**CCG***CAA*AAGAT***TATAGGCAATGGG****CCCATTGCCTATA*CAACAAACT**	-15.50	-14.92	-46.92	-7.04	-52.48
	Truncated amplicon: *hlyA*-3	**TA**CGG**TGGCT**C***CGCA*AAAGAT***GTTGTATAGGCAATGGG****CCCATTGCCTATACAACA*AACT**	-23.63	23.63	-63.18	-7.04	-69.55

	Truncated amplicon: *hlyA*-4	TACGGTGGCTCCGCAAAAGAT*GTTTGTTGTATAGGCAATGGG*CCCATTGCCTATACAACAAACT	-28.14	-26.86	-72.20	-7.04	-80.01
M. leprae	native ssDNA (148 pb)	ATTTCTGCCGCTGGTATCGGTGTCGGCGGCTTGTTGACCGGCCCTCAGCCAGCAAGCAGGCATGCCGCCGGGTGCAGCAGTATCGTGTTAGTGAACAGTGCATCGATGATCCGGCCGTCGGCGGCACATACGGCAACCTTCTAGCGCA	-35.34	-25.64	-81.59	-19.64	-13.62
	Truncated amplicon: *RLEP*-1	GTCGGCGGCTTGTTGACCGGC*GTGTTAGTGAACAGTGCATC	-10.01	-7.96	-26.06	-9.75	-7.05
	Truncated amplicon: *RLEP*-2	GTCGGCGGCTTGTTGACCGGC*TTTTT*GTGTTAGTGGACAGTGCATC	-4.55	-2.55	-15.88	-9.75	-7.05
	Truncated amplicon: *RLEP*-3	GTCGGCGGCTTGTTGACTGGC*TTTTT*GTGTTAGTGGACAGTGCTTC	-3.45	-2.55	-14.50	-4.89	-3.30
L. amazonensis	native ssDNA (222 pb)	ACGAAATAGGAAGCCAAGTCATCCATCGCGACACGTTATGTGAGCCGTTATCCACACACGCACCCCCCCGCCCCAAAAACGGAAAACGCCGTATTGAAACGGGCATTTTTCTCTGCTTTTGTATATACGCGGTTGTGCGCTATAACGTCGATCGGCCATTTTTTGTTTACTGCAAATGTTGTTTTTGAGTACAAAAACTTTGCTGTGTATGTGTGGGAAAGG	-44.73	-30.11	-99.31	-10.68	-8.98
	Truncated amplicon: *ITS1*-1	TCCATCGCGACACGTTATGT*CTGTGTATGTGTGGGAAAGG	-5.37	-3.03	-16.92	-13.51	-1.60
	Truncated amplicon: *ITS1*-2	TCCATCGCGACACGTTATGTGATGGACTGTGTATGTGTGGGAAAGG	-7.08	-5.33	-20.98	-13.51	-1.06
	Truncated amplicon: *ITS1*-3	CGTGTCGCGACACGTTATGT*CTGTGTATGTGTGGGAAAGGAA	-5.31	-5.25	-26.86	-30.01	-1.60
	Truncated amplicon: *ITS1*-4	TCCATTGCGACACGTTATGT*CTGTGTATGTGTGGGAAAGG	-4.48	-3.03	-12.48	-6.30	-1.60

Supplementary Table S2. Synthetic truncated oligonucleotides (syn-trunc-ssDNA) derived from the *ITS1* gene, designed to assess the influence of local secondary structures (s-sΔG) on probe hybridization in AF-NALFA. The underlined sequences represent the native probe-binding sites: DP (detection probe, 5') and CP (capture probe, 3'). Non-highlighted nucleotides correspond to synthetic random sequences inserted either at the 3′ end (*L amazonensis ITS1*-5 to *ITS1*-9, affecting CP) or at the 5′ end (*L. amazonensis ITS1*-10 to *ITS1*-14, affecting DP) to induce progressive structural blocking. Nucleotides in larger font indicate the sequences used in the s-sΔG OligoAnalyzer from IDT™. All constructs retain full complementarity to the probes, and an asterisk separates the DP and CP hybridization sites.

**Identification**	**Sequences (5'-3')**	Base Pairs (potentially blocked)	***s-s*ΔG** IDT™
With all bases free for hybridization (reference)	**TCCATCGCGACACGTTATG***TTTTTTTTTTT***CTGTGTATGTGTGGGAAAGG**		
ITS1-5	**TCCATCGCGACACGTTATG*CTGTGTATGTGTGGGAAAGG***TACACAG	7	-9.15
ITS1-6	**TCCATCGCGACACGTTATG*CTGTGTATGTGTGGGAAAGG***ATACACAG	8	-10.63
ITS1-7	**TCCATCGCGACACGTTATG*CTGTGTATGTGTGGGAAAGG***ACACATACACAG	12	-17.22
ITS1-8	**TCCATCGCGACACGTTATG*CTGTGTATGTGTGGGAAAGG***CCACACATACACAG	14	-22.24
ITS1-9	**TCCATCGCGACACGTTATG*CTGTGTATGTGTGGGAAAGG***CTTTCCCACACATACACAG	19	-32.37
ITS1-10	ATAACGTTTTT***TCCATCGCGACACGTTATG*CTGTGTATGTGTGGGAAAGG**	6	-13.51
ITS1-11	ACATAACG***TCCATCGCGACACGTTATG*CTGTGTATGTGTGGGAAAGG**	8	-12.63

ITS1-12	GACATAACGTTTTTT*<u>**TCCATCGCGACACGTTATG**</u>*<u>**CTGTGTATGTGTGGGAAAGG**</u>	9	-15.55
ITS1-13	ACATAACGTGTC*<u>**TCCATCGCGACACGTTATG**</u>*<u>**CTGTGTATGTGTGGGAAAGG**</u>	11	-18.84
ITS1-14	ACATAACGTGTCGCGA*<u>**TCCATCGCGACACGTTATG**</u>*<u>**CTGTGTATGTGTGGGAAAGG**</u>	17	-32.25

Supplementary Table S3. Pairwise statistical comparison of AF-NALFA signal intensities between native *M. leprae RLEP* amplicons and their truncated variants (*RLEP*-1, *RLEP*-2 and *RLEP*-3). Adjusted p-values were obtained using one-way ANOVA followed by Tukey's post-hoc test. ns = non-significant; p < 0.05; p < 0.01; **p < 0.001; **p < 0.0001.

Name	Adjusted p-value	Summary
native ssDNA vs. *RLEP*-1	<0.0001	****
native ssDNA vs. *RLEP*-2	<0.0001	****
native ssDNA vs. *RLEP*-3	<0.0001	****
native ssDNA vs. Blank	0.9994	ns
RLEP-1 vs. *RLEP*-2	0.4349	ns
RLEP-1 vs. *RLEP*-3	<0.0001	****
RLEP-1 vs. Blank	<0.0001	****
RLEP-2 vs. *RLEP*-3	0.0001	***
RLEP-2 vs. Blank	<0.0001	****
RLEP-3 vs. Blank	<0.0001	****

Supplementary Table S4. Pairwise statistical comparison of AF-NALFA signal intensities between native *L. monocytogenes hlyA* amplicons and the *hlyA* truncated constructs (*hlyA*-1 to *hlyA*-4). Adjusted p-values were obtained using one-way ANOVA followed by Tukey's post-hoc test. ns = non-significant; p < 0.05; p < 0.01; **p < 0.001; **p < 0.0001

Name	Adjusted *p*- value	Summary
native ssDNA vs. *hlyA*-1	<0.0001	****
native ssDNA vs. *hlyA*-2	<0.0001	****
native ssDNA vs. *hlyA*-3	<0.0001	****
native ssDNA vs. *hlyA*-4	<0.0001	****
native ssDNA vs. Blank	<0.0001	****
hlyA-1 vs. *hlyA*-2	<0.0001	****
hlyA-1 vs. *hlyA*-3	<0.0001	****
hlyA-1 vs. *hlyA*-4	<0.0001	****
hlyA-1 vs. Blank	<0.0001	****

hlyA-2 vs. *hlyA*-3	<0.0001	****
hlyA-2 vs. *hlyA*-4	<0.0001	****
hlyA-2 vs. Blank	<0.0001	****
hlyA-3 vs. *hlyA*-4	0.7136	ns
hlyA-3 vs. Blank	0.0008	***
hlyA-4 vs. Blank	<0.0001	****

Supplementary Table S5. Pairwise statistical comparison of AF-NALFA signal intensities between native *L. amazonensis ITS1* amplicons and the *ITS1* truncated constructs (*ITS1*-1 to *ITS1*-4). Adjusted p-values were obtained using one-way ANOVA followed by Tukey's post-hoc test. ns = non-significant; $p < 0.05$; $p < 0.01$; **$p < 0.001$; **$p < 0.0001$.

Name	Adjusted *p*- value	Summary
native ssDNA vs. *ITS1*-1	<0.0001	****
Native ssDNA vs. *ITS1*-2	<0.0001	****
native ssDNA vs. *ITS1*-3	<0.0001	****
native ssDNA vs. *ITS1*-4	<0.0001	****
native ssDNA vs. Blank	0.1232	ns
ITS1-1 vs. *ITS1*-2	<0.0001	****
ITS1-1 vs. *ITS1*-3	<0.0001	****
ITS1-1 vs. *ITS1*-4	0.7752	ns
ITS1-1 vs. Blank	<0.0001	****
ITS1-2 vs. *ITS1*-3	<0.0001	****
ITS1-2 vs. *ITS1*-4	<0.0001	****
ITS1-2 vs. Blank	<0.0001	****
ITS1-3 vs. *ITS1*-4	<0.0001	****
ITS1-3 vs. Blank	<0.0001	****
ITS1-4 vs. Blank	<0.0001	****
